# Gene Panel Analysis in a Large Cohort of Patients With Autosomal Dominant Polycystic Kidney Disease Allows the Identification of 80 Potentially Causative Novel Variants and the Characterization of a Complex Genetic Architecture in a Subset of Families

**DOI:** 10.3389/fgene.2020.00464

**Published:** 2020-05-07

**Authors:** Vilma Mantovani, Sofia Bin, Claudio Graziano, Irene Capelli, Raffaella Minardi, Valeria Aiello, Enrico Ambrosini, Carlotta Pia Cristalli, Alessandro Mattiaccio, Milena Pariali, Sara De Fanti, Flavio Faletra, Enrico Grosso, Rachele Cantone, Elena Mancini, Francesca Mencarelli, Andrea Pasini, Anita Wischmeijer, Nicola Sciascia, Marco Seri, Gaetano La Manna

**Affiliations:** ^1^Medical Genetics Unit, S. Orsola-Malpighi University Hospital, Bologna, Italy; ^2^Center for Applied Biomedical Research (CRBA), University of Bologna, Bologna, Italy; ^3^Nephrology, Dialysis and Transplantation Unit, Department of Experimental, Diagnostic and Specialty Medicine (DIMES), S. Orsola-Malpighi University Hospital, Bologna, Italy; ^4^Department of Biological, Geological and Environmental Sciences, University of Bologna, Bologna, Italy; ^5^Medical Genetics Unit, Institute for Maternal and Child Health - IRCCS “Burlo Garofolo”, Trieste, Italy; ^6^Medical Genetics Unit, AOU Città della Salute e della Scienza, Turin, Italy; ^7^Nephrology, Dialysis and Hypertension Unit, S. Orsola-Malpighi University Hospital, Bologna, Italy; ^8^Pediatrics Unit, S. Orsola-Malpighi University Hospital, Bologna, Italy; ^9^Clinical Genetics Service and South Tyrol Coordination Center for Rare Diseases, Department of Pediatrics, Regional Hospital of Bolzano, Bolzano, Italy; ^10^Radiology Unit, S. Orsola-Malpighi University Hospital, Bologna, Italy

**Keywords:** polycystic kidney disease, ADPKD, *PKD1*, *PKD2*, NGS, cystogenes

## Abstract

**Introduction:** Autosomal dominant polycystic kidney disease (ADPKD) is one of the most common inherited disorders in humans and the majority of patients carry a variant in either *PKD1* or *PKD2*. Genetic testing is increasingly required for diagnosis, prognosis, and treatment decision, but it is challenging due to segmental duplications of *PKD1*, genetic and allelic heterogeneity, and the presence of many variants hypomorphic or of uncertain significance. We propose an NGS-based testing strategy for molecular analysis of ADPKD and its phenocopies, validated in a diagnostic setting.

**Materials and Methods:** Our protocol is based on high-throughput simultaneous sequencing of *PKD1* and *PKD2* after long range PCR of coding regions, followed by a masked reference genome alignment, and MLPA analysis. A further screening of additional 14 cystogenes was performed in negative cases. We applied this strategy to analyze 212 patients with a clinical suspicion of ADPKD.

**Results and Discussion:** We detected causative variants (interpreted as pathogenic/likely pathogenic) in 61.3% of our index patients, and variants of uncertain clinical significance in 12.5%. The majority (88%) of genetic variants was identified in *PKD1*, 12% in *PKD2*. Among 158 distinct variants, 80 (50.6%) were previously unreported, confirming broad allelic heterogeneity. Eleven patients showed more than one variant. Segregation analysis indicated biallelic disease in five patients, digenic in one, *de novo* variant with unknown phase in two. Furthermore, our NGS protocol allowed the identification of two patients with somatic mosaicism, which was undetectable with Sanger sequencing.

Among patients without *PKD1*/*PKD2* variants, we identified three with possible alternative diagnosis: a patient with biallelic mutations in *PKHD1*, confirming the overlap between recessive and dominant PKD, and two patients with variants in *ALG8* and *PRKCSH*, respectively.

Genotype-phenotype correlations showed that patients with *PKD1* variants predicted to truncate (T) the protein experienced end-stage renal disease 9 years earlier than patients with *PKD1* non-truncating (NT) mutations and >13 years earlier than patients with *PKD2* mutations. ADPKD-*PKD1*^T^ cases showed a disease onset significantly earlier than ADPKD-*PKD1*^NT^ and ADPK-*PKD2*, as well as a significant earlier diagnosis. These data emphasize the need to combine clinical information with genetic data to achieve useful prognostic predictions.

## Introduction

Autosomal dominant polycystic kidney disease (ADPKD) is one of the most common inherited disorders in humans, with an estimated prevalence between 1/400 and 1/1000 in the general population (Torres et al., 2007). It is characterized by the development and progressive enlargement of renal cysts, which often leads to end-stage renal disease (ESRD) (Gabow, 1993; Grantham et al., 2011). ADPKD is mainly caused by mutation in either *PKD1* (74–85% of patients) or *PKD2* (15–26%) (Rossetti et al., 2007; Barua et al., 2009; Carrera et al., 2016), with *PKD2* variants being relatively more frequent in Japan (Kurashige et al., 2015). Furthermore, mutations in *GANAB* and *DNAJB11* were recently shown to cause polycystic kidney in a small percentage (0.3%) of affected subjects (Porath et al., 2016; Cornec-Le Gall et al., 2018). Patients with *PKD1* mutations usually develop a more severe disease than patients with *PKD2*, with more cysts at an earlier age (Hateboer et al., 1999; Torres et al., 2007), and truncating mutations in *PKD1* lead to earlier ESRD than non-truncating ones (Cornec-Le Gall et al., 2013). Intra and inter familial disease severity is also reported, suggesting a role for environmental factors and the genetic background. Additional genetic defects are sometime identified in rare cases with early onset disease, i.e., mutations in the second PKD allele, or in other genes such as *PKHD1* or *HNF1B* (Rossetti et al., 2009; Bergmann et al., 2011; Audrézet et al., 2016). Somatic mosaicism and hypomorphic alleles contribute to further increase the genetic complexity of this condition (Connor et al., 2008; Rossetti et al., 2009; Vujic et al., 2010).

Although the diagnosis of ADPKD is usually based on imaging studies, a genetic test is currently indicated in patients without a positive family history, with atypical clinical or imaging features, and in helping the identification of a familial donor for renal transplantation. In addition, a genetic diagnosis may guide clinical management and treatments, and it may improve the accuracy of genetic counseling. Genetic analysis is not uniformly required by nephrologists in standard clinical practice. However, information on specific mutations may help to predict disease progression using the PROPKD score, which can guide the selection of patients who will best benefit from the use of Tolvaptan, a vasopressin 2 receptor antagonist (Cornec-Le Gall et al., 2016, 2019).

The cost of analysis and technical challenges prevented a widespread application of *PKD1* sequencing. This gene has six pseudogenes with high sequence homology, in a duplicated region adjacent to the original *PKD1* locus. In addition, its large size and high GC content, the extensive allelic heterogeneity of *PKD1* (and *PKD2* as well) and genetic heterogeneity further complicate the molecular screening. Next-generation sequencing (NGS) technologies revolutionized the approach to molecular diagnostics and have recently been applied to ADPKD as well, with different strategies. Targeted re-sequencing was performed by generating long-range (LR) locus-specific PCR amplicons and multiplexing them from individuals samples as bar-coded libraries for NGS (Rossetti et al., 2012; Tan et al., 2014; Kinoshita et al., 2016). An alternative approach was a sequence capture-based NGS, with improved algorithms in order to overcome unspecific capture of pseudogenes (Trujillano et al., 2014; Eisenberger et al., 2015). A long-read single-molecule sequencing was recently suggested as a strategy to overcome *PKD1* complexities (Borràs et al., 2017). Finally, a comprehensive approach with 140 candidate gene-panel for cystic and glomerular nephropathies has been proposed (Bullich et al., 2018). All these methods have strengths and pitfalls, and should be further validated.

Here, we propose an NGS-based testing strategy for molecular diagnosis of ADPKD and its phenocopies, validated in a diagnostic setting. Our workflow included LR-PCR targeted re-sequencing for both *PKD1* and *PKD2*, combined with a specific alignment pipeline for *PKD1*. Large rearrangements were assessed by multiplex-ligation probe amplification (MLPA). Additional cystogenes, namely *GANAB, DNAJB11, LRP5, PMM2, PRKCSH, SEC63, SEC61B, ALG8, PKHD1, DZIP1L, HNF1B, UMOD, INF2*, and *REN* (Porath et al., 2016; Cornec-Le Gall et al., 2017; Bullich et al., 2018), were also tested by NGS targeted re-sequencing in *PKD1*/*2* negative patients, after clinical revision.

## Materials and Methods

### Patient Recruitment

Most patients were recruited from the Units of Nephrology and Medical Genetics of Bologna University Hospital, a few from partner centers. Written informed consents for genetic analysis and publication of results were obtained from the patients or their parents/guardians in compliance with national ethics regulation. The families received pre- and post-test genetic counseling.

The 212 patients enrolled in the study belonged to the following three groups: (1) a validation cohort included 21 ADPKD patients that had previously undergone genetic diagnosis by Sanger sequencing of *PKD1* and *PKD2*; (2) a group of 36 patients with a clinical diagnosis of severe ADPKD, who had already received a kidney transplantation, was included as a confirmation cohort; (3) a third group was a discovery cohort, including 155 unrelated patients who had been consecutively referred for ADPKD genetic testing from 2014 to 2018. In addition, 143 family members were tested for the possible pathogenic variants detected in the probands.

Main clinical features of patients submitted to the study are summarized in Table 1, while the most significant clinical histories are extrapolated and described in the Results section.

**Table 1:** Clinical characteristics of study patients.

	***N*.**	**%**
Index patients	212	
Italians	190	89.6
Women	113	53.3
Positive family history (*n* = 193)	148	76.7
Presence of liver cysts (*n* = 159)	128	80.5
Vascular abnormalities (*n* = 121)	24	19.8
Urological events before 35 yr (*n* = 128)	34	26.6
Antihypertensive treatment before 35 yr (*n* = 133)	54	40.6
ESRD (*n* = 165)	42	25.5
Transplantation (*n* = 177)	45	25.4
ADPKD^VEO^ (*n* = 163)	7	4.3
ADPKD^EO^ (*n* = 163)	23	14.1
		**Median value**
Age at test, yr (*n* = 212)		50
Age at onset, yr (*n* = 163)		25
Age at diagnosis, yr (*n* = 156)		41
Serum creatinine at presentation, mg/dl (*n* = 155)		1.15
GFR at presentation, ml/min (*n* = 131)		66

*The number of cases for which the data was available is shown in brackets. ESRD, End-Stage Renal Disease; GFR, Glomerular Filtration Rate; VEO, Very Early Onset (diagnosed in utero or before 18 months); EO, Early Onset (diagnosed before 15 years)*.

The diagnosis of ADPKD relies primarily on imaging, although some cases are diagnosed by genetic testing. Typical ultrasonography imaging reveals large kidneys with multiple bilateral cysts. Imaging is also the first choice for pre-symptomatic diagnosis of patients with a positive family history. Specifically, Pei's criteria suggest that the presence of a total of three or more kidney cysts for at-risk individuals aged 15–39 years and 2 or more cysts in each kidney for at-risk individuals aged 40–59 years are sufficient for a clinical diagnosis. The absence of kidney cysts on ultrasound excludes ADPKD in patients older than 40 years. If ultrasonography images are equivocal, magnetic resonance imaging (MRI) or computed tomography (CT) may clarify the diagnosis (Pei et al., 2009).

### Library Preparation

Genomic DNA was isolated from EDTA peripheral blood using the semi-automatic Maxwell 16 instrument (Promega Corporation, Madison, WI USA).

The *PKD1* (MIM#601313 HGNC:9008, RefSeq NM _001009944.2) entire coding region, including exon-intron boundaries (at least 50 bp) and most of the 5′ and 3′ untranslated regions were amplified in eight long-range (LR) PCR using specific primers anchored on the rare mismatch sequencing between *PKD1* and pseudogenes. We combined previously described primers (Audrézet et al., 2012; Tan et al., 2014; Kinoshita et al., 2016) with primers designed by us and we optimized the PCR protocols. A LR-PCR method was validated also for *PKD2* (MIM#173910 HGNC:9009, RefSeq NM_000297.3), using previously described primers (Tan et al., 2014). Primers and PCR conditions are reported in Supplementary Table 1.

For each patient, all 13 LR-PCR products (37.8 kb for *PKD1* and 35.9 kb for *PKD2*) were pooled together in equimolar manner (50 pM each), the pools were then subjected to enzymatic digestion using Ion Xpress Plus Fragment Library Kit, in order to obtain about 200 bp fragments. Fragmented LR-PCRs were used to construct barcoded libraries using Ion Xpress Barcode Adapters following the manufacturer's protocol. A single barcode was used for simultaneous analysis of both genes for each patient. Size selection of fragments was performed by E-Gel Size Select II 2% agarose gel (Thermo Fisher Scientific Inc.).

Patients without *PKD1*/*PKD2* genetic defects were revised by clinicians and, if appropriate, were tested for mutations in additional genes. To this aim, we developed a multiplexed PCR NGS for a panel including the novel described ADPKD genes (*GANAB*, MIM#600666 HGNC:4138 RefSeq NM_198335.3*; DNAJB11*, MIM#611341 HGNC:14889 RefSeq NM_016306.5), the autosomal dominant polycystic liver disease (ADPLD) genes (*LRP5*, MIM#603506 HGNC:6697 RefSeq NM_002335.3; *PMM2* MIM#601785 HGNC:9115 RefSeq NM_000303.2; *PRKCSH*, MIM#177060 HGNC:9411 RefSeq NM_002743.3; *SEC63*, MIM#608648 HGNC:21082 RefSeq NM_007214.4; *SEC61B*, MIM#609214 HGNC:16993 RefSeq NM_006808.2; *ALG8*, MIM#608103 HGNC:23161 RefSeq NM_024079.4), the two genes for ARPKD (*PKHD1*, MIM#606702 HGNC:9016 RefSeq NM_138694.3 and *DZIP1L*, MIM#617570 HGNC:26551 RefSeq NM_173543.2), and additional cystogenes (*HNF1B*, MIM#189907 HGNC:11630 RefSeq NM_000458.3; *UMOD*, MIM#191845 HGNC:12559 RefSeq NM_001008389.2; *INF2*, MIM#610982 HGNC:23791 RefSeq NM_022489.3; *REN*, MIM#179820 HGNC:9958 RefSeq NM_000537.3). Amplicon-based libraries were generated from 10 ng of DNA per sample using Ion AmpliSeq Library Kit Plus according to the manufacturer's instructions (Thermo Fisher Scientific Inc.).

### Template Generation and Sequencing

The barcoded libraries were quantified using Ion Library TaqMan Quantitation Kit and pooled in equimolar manner (9 pM), then the emulsion PCR was performed using the Ion OneTouch 2 System (Thermo Fisher Scientific Inc.). The enriched emulsion-PCR was then prepared for sequencing protocol using Ion PGM Hi-Q Sequencing Kit, loaded onto an Ion 318v2 chip and sequenced with the Ion Personal Genome Machine (PGM). For *PKD1* and *PKD2* genes, 12 patients were simultaneously analyzed in each chip. Recently, we introduced the HID Ion Chef instrument for emulsion PCR and enrichment, united with Ion Gene Studio System S5 (Thermo Fisher Scientific Inc). We simultaneously analyzed 16 patients on an Ion 520 chip. For the other genes, the number of patients per chip has been calculated to obtain an average cover of at least 500x.

### Data Analysis

The raw sequencing data were transferred to the Torrent Server where the Torrent Suite^TM^ performed the alignment to a reference genome in order to generate Fastq files, Binary Alignment Map (BAM) in conjunction with Binary Alignment Index (BAI) and Variant Call Format (VCF) files. *PKD1* reads were aligned against a modified reference genome based on chromosome 16 of Human Genome 38 (Grch38), where all the nucleotides outside the *PKD1* locus were masked and replaced with “Ns.” All the VCF files were uploaded into Ion Reporter software (Thermo Fisher Scientific Inc.) selecting the Annotation Variant workflow in order to associate to each variant the nucleotide change in mRNA transcript, the aminoacidic change, the exons or IVSs and the function.

The BAM/BAI files, generated following the alignment, were visualized using Integrative Genome Viewer (IGV) software^1^. IGV was used to assess the depth of coverage of the sequencing reads, zygosity, quality of the sequencing reads and the mapping quality.

### Filtering and Classification of the Gene Variants

Variant filtering based on population frequency was performed using population databases ExAC, gnomAD (Lek et al., 2016), 1000Genomes (1000 Genomes Project Consortium et al., 2015) and dbSNP database to include only rare alleles (minor allele frequency ≤1%).

The variants were then annotated according to the guidelines published by the Human Genome Variation Society^2^ (Den Dunnen and Antonarakis, 2000) and classified in five categories, according to the American College of Medical Genetics and Genomics (ACMG) standards (Richards et al., 2015). Segregation analysis was performed in additional affected family members whenever possible. The protein truncating (PT) mutations (frameshift, non-sense, canonical splice-site and large-gene rearrangements) were considered pathogenic (P). The non-truncating (NT) variants (in-frame insertion/deletion, non-synonymous and synonymous variants) were attributed to one of the other four categories, by using the VarSomeClinical platform^3^. We also checked variants in mutation database PKDB^4^, in Leiden Open Variations PKD Database^5^, in Human Gene Mutation Database (Stenson et al., 2017), in ClinVar archive^6^ and by segregation analysis, and we re-classified them when appropriate.

In addition to the five categories defined by ACMG standards, we classified some PKD variants as hypomorphic (H), according to the literature or databases. We also re-classified H some non-truncating *PKD1* alleles showing incomplete penetrance through family study.

Missense variants predicted to be neutral, rare synonymous and intronic variants predicted as not affecting function, deep intronic and frequent synonymous variants in non-conserved sequence domains were classified likely benign (LB) or benign (B) and are not reported.

We performed an additional bioinformatics analysis for missense variants of uncertain significance (VUS) and for H variants by using PyMOL, an open source molecular graphics system 2.0 Schrodinger, LLC^7^. Five variants in *PKD1* and two variants in *PKD2*, lying in the crystalized portion of the proteins (PDB number: 6A70), were submitted to the tool.

### Sanger Sequencing and MLPA

All the variants considered as pathogenic, likely pathogenic and VUS were confirmed by Sanger sequencing on 3730 DNA Analyzer (Applied Biosystem, Foster City, CA, USA). Coding and splicing regions with low coverage (<20x) have also been sequenced by the Sanger method. For the exons 1-33 of *PKD1*, we performed a nested PCR of the interested region from LR amplicons before proceeding to Sanger sequencing.

Samples without variants classified as P or LP in *PKD1/PKD2*, including those presenting a VUS, were analyzed by MLPA for detection of large gene rearrangements, using commercially kits SALSA MLPA P351 *PKD1* and SALSA MLPA P352 *PKD1*-*PKD2* probemix (MRC-Holland, Amsterdam, NL). Fragment analysis of the PCR product was carried out on 3730 DNA Analyzer and the electropherograms were analyzed by Coffalyzer software (MRC Holland, Amsterdam, NL).

### Statistical Analyses

Continuous variables were reported as mean if normally distributed and median if not, and discrete variables were reported as percentages. The statistical significance has been tested by Pearson's Goodness of Fit Chi-square for categorical variables. Fisher Exact test has been applied in the case of a value less than five in one of the cells. One-way analysis of variance was performed for continuous traits measurement of central tendency. We performed the analysis using the Statistix 8 software (Analytical Software, FL, USA).

## Results

### Identification of *PKD1* or *PKD2* Genetic Defects in Probands

The NGS performed on LR-PCR libraries for *PKD1* and *PKD2* showed an excellent read depth, providing an average base coverage of 1,156x without gaps. On average, 92% of target sequences were covered >20x, but the regions with lower coverage were mainly in deep introns. The exon 1 of *PKD1*, usually difficult to sequence due to its high GC content, resulted entirely sequenced, with an average coverage at 100x.

We adopted the recently suggested nosology, describing the disease and the genetic etiology of ADPKD, classifying the patients as ADPKD-*PKD1*^T/NT^ or ADPKD-*PKD2*, as shown in Table 2 (Eckardt et al., 2015; Cornec-Le Gall et al., 2017). Patients harboring more than one variant were classified ADPKD-PKD1/2^T/NT^ based on the main variant.

**Table 2:** Patient groups and positives for pathogenic/likely pathogenic variants in *PKD1/PKD2*.

	**All (*****n*** **=** **212)**		**Validation cohort (*****n*** **=** **21)**		**Confirmation cohort (*****n*** **=** **36)**		**Discovery cohort (*****n*** **=** **155)**	
	***n***	**%**	***n***	**%**	***n***	**%**	***n***	**%**
*PKD1/PKD2* Positive	138	65.1	21	100.0	31	86.1	86	55.5
ADPKD-*PKD1*	118	85.5	18	85.7	28	90.3	72	83.7
ADPKD-*PKD1^*T*^*	92	78.0	12	66.7	25	89.3	55	76.4
ADPKD-*PKD1^*NT*^*	26	22.0	6	33.3	3	10.7	17	23.6
ADPKD-*PKD2*	20	14.5	3	14.3	3	9.7	14	16.3
*PKD1/PKD2* Negative	74	34.9	0	0.0	5	13.9	69	44.5
*PKD1/2 ^*VUS*^*	24	11.3	0	0.0	3	8.3	21	13.5
ADPKD-*PKD1^*VUS*^*	21	87.5	0	0.0	3	100.0	18	85.7
ADPKD-*PKD2^*VUS*^*	3	12.5	0	0.0	0	0.0	3	14.3

*One proband, homozygous for a hypomorphic PKD1 variant, is also included among the positives. Patients showing variants of uncertain significance (VUS) are also reported and included among the negatives. T, protein truncating; NT, protein non-truncating*.

To validate our method, LR-PCR re-sequencing was performed on DNA from 18 ADPKD-*PKD1* and 3 ADPKD-*PKD2* patients who had previously undergone genetic diagnosis by Sanger sequencing. All the 21 potentially causative variants known to be present in *PKD1* (9 missense, 4 non-sense, 4 splicing and 4 frameshift), as well as the three in *PKD2* (2 frameshift and 1 missense), were correctly detected.

In order to confirm the accuracy of the method, we tested 36 additional patients with a clinical diagnosis of ADPKD, who had already undergone kidney transplantation. In 28 individuals we identified possible pathogenic variants (P or LP) in *PKD1*, 25 of which were T and three NT. In three cases, protein truncating variants in *PKD2* were detected. Three patients showed a variant of uncertain significance in *PKD1*. The low prevalence of *PKD2* mutations (9.7%) compared to *PKD1* (90.3%) in this cohort was consistent with the disease severity in these patients.

We then applied the method to a group of 155 newly recruited patients (discovery cohort). Among these cases, composed of patients with varying degrees of disease severity, 72 patients resulted positive for potentially causative variants in the *PKD1* gene, 55 T and 17 NT, whereas 14 patients showed genetic defects in *PKD2* gene. In 21 cases, we identified a variant classified as VUS, 18 in *PKD1* and three in *PKD2*. In this discovery cohort, the prevalence of *PKD2* mutations (16.3%) is higher when compared to the confirmation cohort.

Overall, in confirmation and discovery cohorts, we detected causative variants in 117 out of 191 patients, obtaining a diagnostic rate of 61.3%; 85.5% of positives showed variants in *PKD1* gene, whereas 14.5% showed variants in *PKD2*. Diagnostic rates are heterogeneous among different studies and can vary in relation to the criteria for variant classification and the proportion of typical/familial cases. A diagnostic rate of 80% was reported in the only large Italian cohort of ADPKD patients, including “highly likely” and “likely” pathogenic variants (Carrera et al., 2016), but studies in other European populations report lower diagnostic rates (Hoefele et al., 2011; Obeidova et al., 2014).

### Accuracy of the Method and Mutation Spectrum

Since we re-sequenced all the possible pathogenic variants by Sanger method, we have been able to calculate sensitivity, specificity and accuracy of NGS, using the Sanger as gold standard. Our NGS on LR-PCR for *PKD1* and *PKD2* genes provided 100% sensitivity, 63% specificity and 92% accuracy. Although we cannot rule out that some variants have not been called by NGS, we have not recorded false negative. On the other hand, detection of false positive variants was not so rare (22/221, 10%) and usually involved GC reach regions and/or homopolymers. However, the frequencies of the two called alleles in these cases was usually unbalanced (Supplementary Table 2).

Two cases of allele-drop out of the wild-type allele were detected, using LR-PCR amplification of exons 13–21 of *PKD1*. In both cases, replacing the LR-PCR primers with those previously described by Rossetti et al. (2012) for exons 15–21, the heterozygous state of the mutations was called correctly. For this reason, we decided to implement our analysis with this last LR-PCR, to avoid possible pitfalls (Supplementary Table 1).

Types and numbers of the variants classified in the P, LP, VUS, and H categories are listed in Table 3. Among the 158 different genetic alterations detected in our study, 80 (50.6%) had not been previously described, 73 of which were found in *PKD1* and 7 in *PKD2*. Missense and non-sense were the most frequent variants in both genes, followed by frameshift in *PKD1* and by splicing and frameshift in *PKD2*. Six variants in *PKD1* occurred *de-novo*, three of which were not previously described: c.3236del p.(Asp1079Alafs^*^25), c.8860G>T p.(Glu2954^*^), and c.9201+1G>A. One *de-novo* and novel variant was also detected in *PKD2*: c.992G>A p.(Cys331Tyr).

**Table 3:** Types of 158 distinct possible pathogenic variants detected in *PKD1* and *PKD2* genes.

			***PKD1*** **variants**		***PKD2*** **variants**	
	***N***	**%^A^**	***N***	**%^A^**	***N***	**%^A^**
All variants	158	100.0	139	88.0	19	12.0
			***N***	**%^B^**	***N***	**%^C^**
Novel	80	50.6	73	52.5	7	36.8
Known	78	49.4	66	47.5	12	63.2
*De novo*	7	4.4	6	4.3	1	5.3
Truncating	100	63.3	85	61.2	15	78.9
Non-truncating	58	36.7	54	38.8	4	21.1
Pathogenic	95	60.1	82	59.0	13	68.4
Likely pathogenic	25	15.8	21	15.1	4	21.1
VUS	30	19.0	28	20.1	2	10.5
Hypomorphic	8	5.1	8	5.8	0	0.0
Missense	54	34.2	50	36.0	4	21.1
Non-sense	46	29.1	41	29.5	5	26.3
Frameshift/indel	36	22.8	32	23.0	4	21.1
Splicing	10	6.3	6	4.3	4	21.1
Large rearrangements	8	5.1	6	4.3	2	10.5
In-frame indel	4	2.5	4	2.9	0	0.0

A % among all variants;

B % among PKD1 variants;

C *% among PKD2 variants. Pathogenic, Likely pathogenic and VUS are classified following American College of Medical Genetics and Genomics standard*.

All the 158 variants detected in *PKD1* and *PKD2* genes that we classified as P, LP, VUS, and H are listed in Supplementary Table 3, whereas the variants classified as LB and B are not reported. The mutations were spread over almost all exons, without hot spot regions. Seventeen mutations were detected in two or more families not known to be related.

Ten index patients showed large rearrangements (6 in *PKD1* and 4 in *PKD2*), four of which were not previously described. These rearrangements could be detected by MLPA only, whereas our NGS analysis was unable to reveal them with enough accuracy. Among *PKD1* deletions, we detected one single whole gene deletion, a deletion of exon 40, and four intragenic multiple exon deletions. In *PKD2*, a deletion of the whole gene was detected in two patients, whereas the deletion of exons 8 and 9 was shown in other two cases.

The molecular visualization of uncertain and hypomorphic variants performed by PyMol revealed alteration in only two of them. After searching for hydrogen bonds within 5 residues around the variants, we detected a bond loss for aminoacid changes p.(Ala3958Pro) (previously undescribed and classified as LB according to ACMG) and p.(Arg3277Cys) (previously reported as H by Rossetti et al., 2009, but classified as LB by ACMG) in Polycystin-1, as shown in Supplementary Figure 1. These results suggest a possible pathogenic role for these variants. Based on this prediction and thanks to segregation analysis we re-classified these two variants as VUS and H, respectively.

### Genotype-Phenotype Analyses

The relationship between affected genes and type of mutation with main clinical features is presented in Supplementary Table 4. A significant association was detected with *PKD1* truncating, *PKD1* non-truncating and *PKD2* mutations vs. kidney transplantation (*p* = 0.0006), antihypertensive treatment before 35 years (*p* = 0.0008), urological events before 35 years (*p* = 0.02).

A significantly different mean age distribution of disease onset (*p* = 0.0004), diagnosis (*p* = 0.0008) and ESRD (*p* = 0.04) was found among the cases with mutations in *PKD1* and *PKD2*. Similar results were obtained by comparing the patients with *PKD1* truncated mutations, *PKD1* non-truncated and *PKD2* mutations. ADPKD-*PKD1*^T^ patients had an onset of the disease, respectively >10 years and >19 years earlier than ADPKD-*PKD1*^NT^ and ADPKD-*PKD2*, as well as a 7 and 16 years earlier diagnosis, respectively. Moreover, ADPKD-*PKD1*^T^ patients experienced ESRD 9 and >13 years earlier than ADPKD-*PKD1*^NT^ and ADPKD-*PKD2*, respectively. These genotype-phenotype correlations are in line with previously published cohorts (Cornec-Le Gall et al., 2013; Hwang et al., 2016).

We found that 23.3% of our patients were without an apparent family history of polycystic kidney disease. Mutations in *PKD1* or *PKD2* were detected in 31 (68.9%) and five (16.1%) of these cases, respectively (Table 4). We demonstrated that the mutation arose *de-novo* in three patients, *one* case showed a somatic mosaicism, and five patients were carriers of two variants, partially explaining the absence of family history for the disease. The remaining 14 patients without family history, resulted negative for *PKD1* or *PKD2* mutations. Among them, after clinical revision, 11 were submitted to additional cystogenes re-sequencing and one of them resulted mutated in both allele of *PKHD1*, whereas three were *PKHD1* heterozygous carriers. We did not observe significant clinical differences between patients with positive and negative family history.

**Table 4:** Patients without family history of ADPKD.

	***N***	**%**
Cases without family history of ADPKD	45	23.3
Cases with *PKD1/PKD2* variants	31	68.9
*PKD1^*T*^*	16	51.6
*PKD1^*NT*^*	10	32.3
*PKD2*	5	16.1
Cases with more than one variant	5	16.1
Cases with *de-novo* variants	3	9.7
Mosaicism	1	3.2
*PKD1/PKD2* negative	14	31.1
Tested for other cystogenes	11	78.6
Cases with variants in other genes (PKHD1)	4	28.6

*Family data was available for 193 index cases*.

### Probands With More Than One Variant

Although ADPKD is typically a late-onset disease, some patients show an early and severe phenotype: ADPKD_VEO_ (diagnosed *in utero* or before 18 months) and ADPKD_EO_ (diagnosed before 15 years). Mutations in multiple PKD genes or biallelic PKD mutations can often explain these phenotypes (Bergmann et al., 2011; Audrézet et al., 2016), proving that additional PKD alleles in trans and/or *de novo* exert an aggravating effect and contribute to early and severe disease expression in polycystic kidney disease. In our series, 30 index patients (18.4%) had either a prenatal identification of renal cysts or clinical symptoms that allowed a diagnosis earlier than 15 years of age (Table 1).

Segregation analysis, when performed in patients who were found to carry more than one possible pathogenic variant, demonstrated the presence of compound heterozigosity in 6 and of a *de novo* variant in two (Table 5). Among patients with two variants, there were 4 ADPKD_VEO_ and 3 ADPKD_EO_. Patients who carried two variants in *cis* are not reported.

**Table 5:** Probands with more than one variant.

**ID**	**Main variant**	**Additional variant**	**Phase**	**Affected relative**	**Age at diagnosis**
15686	PKD1: c.2098-2_2109del	PKD1: p.R3247C	uk	None	20
16051	PKD1: p.E3872*	PKD1: p.S2757C	uk	Daughter	42
16533	PKD1: p.D1079Afs*25	PKD1: p.T2250M	*dn*	None	3 (EO)
16813	PKD1: p.Q493*	PKD1: p. R944C	uk	None	uk
17016	PKD1: p.Y1599*	PKD1: p.S2000C	*trans*	Mother	3 (EO)
17045	PKD1: p.R3277C	PKD1: p.R3277C	*trans*	None	22
17469	PKD1: p.A3958P	PKD1: p.P2674S	*trans*	Mother	10 (EO)
17474	PKD1: p.N2167D	PKD1: p.A561V	*dn*	None	Pn (VEO)
18206	PKD1: p.L1479Wfs*55	PKD1: p.I3167F	*trans*	Father	Pn (VEO)
18287	PKD2: p.C331Y	PKD1: p.S123T;	*trans*	Mother	Pn (VEO)
	PKD2: p.R872G				
18477	PKD1: p.R459P	PKD1: p.G1185D	*trans*	Father	Pn (VEO)

The cases who presented the variants in cis are not reported. VEO, Very Early Onset; EO, Early Onset; uk: unknown; dn, de novo; Pn, Prenatal. *stop codon (Ter).

Based on familial segregation or pathogenicity information, these variants were classified as *main* and *additional*. In patients with an early onset of disease, whenever the additional variant was detected in *trans*, we re-classified it as hypomorphic, as it likely contributed to worsening the phenotype. In six families, we were able to diagnose a possible biallelic disease, resulting from the presence of two variants in *trans* (either in the same or different genes).

In four families, one variant was transmitted from a typically affected parent to a child with early onset disease or to an affected pregnancy, and a second hypomorphic variant was transmitted by an unaffected parent. (1) The p.(Ser2000Cys) (absent in GnomAD but with benign computational predictions) was identified in a child with disease onset at 3 years, in *trans* with a truncating mutation. (2) The p.(Pro2674Ser) variant is frequent, but it was identified in a child with early onset disease, in *trans* with a main variant, p.(Ala3958Pro), which was present in her typically affected mother. (3) In family 18206, two consecutive pregnancies were interrupted after sonographic detection of renal cysts. The father had a diagnosis of ADPKD and carried a frameshift mutation; molecular analysis of fetal DNA identified a second variant transmitted by a healthy mother, p.(Ile3167Phe), which was initially defined as possibly pathogenic (Rossetti et al., 2002), but is relatively frequent in the general population; we propose that it may represent a hypomorphic allele. (4) In family 18477, a pregnancy was interrupted after sonographic detection of renal cysts and *PKD1* analysis revealed two variants: p.(Arg459Pro), transmitted by the affected father, and p.(Gly1185Asp), present in the mother, who had no renal cysts; a healthy child was born from a second pregnancy and he was found to carry the p.(Gly1185Asp) only. Although the p.(Arg459Pro) is extremely rare (never reported before, absent in GnomAD), it is likely benign according to ACMG guidelines; we cannot exclude that it might be in *cis* with an unidentified pathogenic variant.

In family 17045, a homozygous missense variant in *PKD1* was detected. The proband showed an equivocal disease presentation and negative family history, but the first clinical hypothesis, at 22 years of age, was ARPKD. As *PKHD1* screening resulted negative, ADPKD genes were studied and homozygosity for the known hypomorphic variant p.(Arg3277Cys) was identified (Rossetti et al., 2009). Interestingly, PyMol analysis, as well additional bioinformatics tools, predicted a possible pathogenicity for this variant. MLPA did not identify any deletion and no consanguinity was recorded.

Two patients showed ADPKD_EO_ and ADPKD_VEO_, respectively, and both were carriers of a *de novo* P/LP *PKD1* variant and a second heterozygous variant. Patient 16533 was a 15 years old boy who showed an episode of macroematuria at the age of three; abdominal ultrasound showed multiple cysts in both kidneys and the number of cysts and total kidney volume progressed over time. Family history was negative and he was found to carry a *de novo* pathogenic (frameshift) mutation and the p.(Thr2250Met) variant, inherited from his healthy father. The latter variant is relatively frequent in the general population and was already reported as a possible hypomorphic allele in previous studies (Irazabal et al., 2011). In family 17474, renal cysts were detected prenatally and confirmed after birth, parents are healthy (no cysts detected at renal ultrasound) and non-consanguineous. *PKD1* sequencing identified a likely pathogenic variant, p.(Asn2167Asp), absent in parents, and a second maternally inherited variant, p.(Ala561Val). This is extremely rare (never reported before, absent in GnomAD), but with benign computational predictions, and it was classified as hypomorphic. We cannot formally test if variants in these two patients are in *trans*, but we presume they contributed to the severe clinical expression.

**Figure 1:**
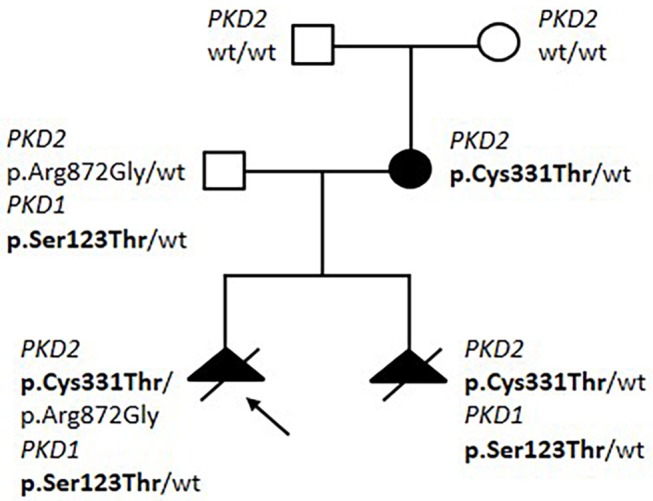
Pedigree of Family 18287 with bilineal inheritance of *PKD1/PKD2* variants. Variants segregating with disease are shown in bolt.

In family 18287 we detected a possible bilineal inheritance, with variants in both *PKD1* and *PKD2* (Figure 1). Two pregnancies were interrupted due to a prenatal finding of polycystic kidney disease at ultrasound examination at 20 and 13 gestational weeks, respectively. The mother was 33 year old; she had multicystic bilateral disease without affected family members, and showed a *de novo* missense variant p.(Cys331Thr) in *PKD2*. The father was a healthy 44 years old man with no signs of kidney cystic disease at ultrasound, and showed a variant in *PKD1*, p.(Ser123Thr), and a second variant in *PKD2*, p.(Arg872Gly). Both fetuses inherited the maternal *PKD2* missense variant, in addition to the paternal p.(Ser872Gly) variant in *PKD1*, while only one fetus inherited the p.(Arg872Gly) *PKD2* variant. The analysis of *PKHD1* performed on the first fetus showed no mutations. We suggest that: 1) the *PKD2* p.(Cys331Thr) variant is pathogenic, since it is *de-novo* in a patient with a renal cystic disease and is transmitted to both fetuses; 2) the p.(Ser123Thr) variant in *PKD1* is hypomorphic, since it does not cause renal disease in the father (age 44 years), but worsens the renal phenotype when co-inherited with a *PKD2* mutation; 3) the missense variant p.(Arg872Gly) in *PKD2*, already described as disease-causing in HGMD database (Neumann et al., 2013), is likely benign, since it is present in a healthy man and does not segregate with disease in the fetuses.

Bilineal inheritance of *PKD1* and *PKD2* variants is an extremely rare finding and, as expected, disease associated with the presence of two distinct mutations appeared to be more severe than the disease associated with either mutation alone (Pei et al., 2001; Elisakova et al., 2018).

### Detection of Mosaicism

The high sensitivity of NGS technology allowed us to detect two cases of mosaicism.

**Figure 2:**
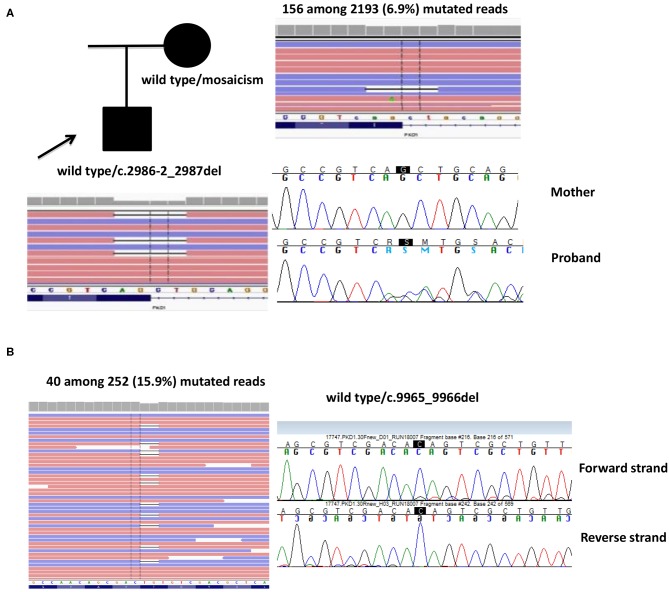
Two cases of mosaicism. **(A)** Family 16321, where the affected mother showed 6.9% of mutated *PKD1* in peripheral blood. **(B)** Family 17747, where the proband showed 15.9% of mutated *PKD1*. The reads are visualized by Integrative Genome Viewer (IGV) software.

**Case 1**. A 53-year-old male proband was first evaluated at the age of 34, when he had an episode of hematuria. Abdominal US documented bilateral multiple renal cysts without hepatic involvement. He started treatment for arterial hypertension at the age of 40 years and he underwent to hemodialysis at 48 years old. He received a kidney transplant 2 years later. The molecular analysis showed the heterozygosis for c.2986-2_2987del splicing mutation in *PKD1*: this four-nucleotide deletion between intron12 and exon13 was predicted to alter an acceptor site, affecting splicing. The proband's mother was also diagnosed with ADPKD at the age of 67 years, when she performed an ultrasound for abdominal pain, although she showed a milder phenotype (renal and hepatic cysts but normal renal function at 69 years) and no other family members were reported to be affected. The c.2986-2_2987del mutation was not detected by targeted Sanger sequencing in the mother. NGS of whole *PKD1* was later performed on maternal DNA and the mutation c.2986-2_2987del was detected in 156 among 2,193 reads, revealing a 6.9% of mosaicism in peripheral blood. Of note, the mutation was not sufficient for the germinal variant calling (threshold defined by our bioinformatics pipeline was 10%), but it was readable by IGV software (Figure 2A).
**Case 2**. A 63-year-old female patient, diagnosed at 46 years and without familiarity for the disease, showed no heterozygous mutations or rearrangements in *PKD1* and *PKD2* genes. However, a 2bp deletion (c.9965_9966del) in exon 30 of *PKD1* gene was detected in 40 among 252 (15.9%) reads. Sanger sequencing by using two different gene-specific LR-PCR primers pair and two different sequencing primers pair confirmed the presence of the mosaicism (Figure 2B).

### Patients Without *PKD1* and/or *PKD2* Mutations

In 74 index patients, neither pathogenic/likely pathogenic variants nor rearrangements were identified in both *PKD1* and *PKD2*, although 24 of them showed a VUS. Family data were recorded for 42 cases, and 28 had a family history of polycystic disease. Among families, disease progression was typical in at least eight, while it was very mild and/or atypical (based on imaging studies) in the remaining 14 families. The median age of disease onset was 30 years; six patients had reached ESRD and three underwent renal transplantation. After re-evaluation of the clinical phenotype, 28 of these patients were tested for an additional NGS-panel that included the recently described genes associated with ADPKD and ADPLD, *PKHD1* and some additional cystogenes. Six patients showed variants in one or more of these genes, as reported in Table 6.

**Table 6:** Molecular defects in additional cystogenes detected in 25 *PKD1/PKD2*-negative cases.

**ID**	**Age at diagnosis**	**Gene**	**Exon**	**Protein**	**cDNA**	**ACMG classification**
16542	42	*PKHD1*	58	p.H3124Y	c.9370 C>T	VUS
		*PMM2*	2	p.G42R	c.124 G>A	LP
16672	41	*PKHD1*	66	p.R3913C	c.11737C>T	VUS
17254	15	*PKHD1*	5	p.R124*	c.370C>T	P
17316	17	*PKHD1*	3	p.T36M	c.107C>T	LP
		*PKHD1*	46	p.C2422G	c.7264T>G	LP
17654	61	*ALG8*	4	p. L149R	c.446T>G	VUS
18607	59	*PRKCSH*	15	p.Y423*	c.1269C>G	LP

ACMG, American College of Medical Genetics and Genomics standard. *stop codon (Ter).

**Figure 3:**
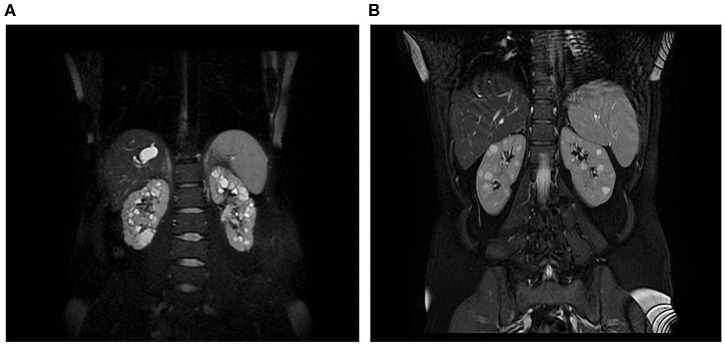
Kidney and liver images of two patients. **(A)** MRI of a 31-year-old woman with compound heterozygous mutations in *PKHD1*, who received a diagnosis of polycystic kidneys at 17. **(B)** MRI of a 45-year-old woman, who carried two heterozygous variants in *PKHD1*, p.(His3124Thr), and *PMM2*, p.(Gly42Arg).

Patient 17316 carried compound heterozygous mutations in *PKHD1*, hence a diagnosis of ARPKD could be posed. She was a 31 years old woman who received a diagnosis of polycystic kidneys at 17. At 30, after her first pregnancy, she had proteinuria, multiple cysts in both kidneys and a mild reduction of GFR. No family members were reported to be affected by polycystic kidney and ADPKD gene testing resulted negative. An abdominal computed tomography scan showed, in addition to the known renal cysts, segmental dilatation of the intrahepatic bile ducts compatible with Caroli disease. *PKHD1* analysis identified two mutations in compound heterozygosity, p.(Thr36Met) and p.(Cys2422Gly). MRI is shown in Figure 3A.

Three additional patients showed heterozygous variants in *PKHD1* (MLPA excluded large intragenic deletions/duplications). Monoallelic mutations in *PKHD1* have been described to cause multiple liver cysts and/or increased kidney echogenicity in up to 10% of carriers, but were not associated with an increased risk of renal cysts (Gunay-Aygun et al., 2011). Patient 17254 carried a definitely pathogenic variant, p.(Arg124^*^): she was a 50 year old woman, renal cysts were identified when she was 15, but the disease was non progressive, she had no hepatic cysts and no family history. The *PKHD1* mutation likely represents a spurious finding. Also patient 16672, a 42 year old man, found out to have renal cysts at the age of 40, together with multiple hepatic cysts; he carried a heterozygous variant of unknown significance, p.(Arg3913Cys).

Finally, patient 16542 was diagnosed with PKD at the age of 30 years, after the onset of arterial hypertension (MRI is shown in Figure 3B); she had normal renal function at last evaluation (45 years). Her father died at the age of 79, he had type 2 diabetes requiring insulin treatment, renal and hepatic cysts detected at ultrasonography and normal renal function. The patient carried a heterozygous variant of unknown significance in *PKHD1*, p.(His3124Tyr), defined as likely pathogenic in ClinVar, and a missense variant p.(Gly42Arg) in *PMM2*, classified as likely pathogenic. Recessive mutations in *PMM2* were reported as associated to hyperinsulinemic hypoglycemia (HI) and PKD (Cabezas et al., 2017). The study of larger cohorts of patients will be able to define if the combination of variants in distinct recessive genes can be relevant for cysts progression.

Patient 17654 was diagnosed with polycystic kidney disease at the age of 61 on routine abdominal US examination. He has renal and hepatic cysts but normal renal function. His 89-year-old mother has renal and liver cysts, and a nephrocalcinosis. An uncle, who died at the age of 85, had kidney cysts and mild renal insufficiency. Molecular analysis showed a variant of uncertain significance in *ALG8*, p.(Leu149Arg), which is extremely rare in the general population and has a pathogenic computational verdict with no benign predictions. *ALG8* was recently described as a very rare cause of ADPLD, but at least one patient had renal cysts at a young age (Besse et al., 2017). In our family, the p.Leu149Arg variant was present in the proband's mildly affected mother. *ALG8* may be the cause of polycystic disease in this family, although a functional assay would be needed to prove the involvement of this variant beyond doubt.

Patient 18607 was diagnosed with polycystic disease at the age of 49, with cysts mainly affecting the liver. Family history was not known, but she carried a non-sense mutation p.(Tyr423^*^) in *PRKCSH*, a gene associated to polycystic liver disease-1 (PCLD1) with or without kidney cysts (Fedeles et al., 2011). The same mutation, p.(Tyr423^*^), was already reported in a family with autosomal dominant polycystic liver disease (Li et al., 2003).

## Discussion

Genetic testing in the clinical management of ADPKD is not widespread for several reasons. First, analysis of *PKD1* is challenging due to its segmentally duplicated region. Second, the genetic heterogeneity and the extreme allelic heterogeneity (>1.500 different pathogenic variants described for *PKD1*) require analysis of the coding regions of all candidate genes, and Sanger-based single gene approaches are costly and cumbersome. Third, the presence of many non-truncating alleles and potentially hypomorphic low-penetrance variants pose a further challenge for clinical interpretation. However, genetic testing is increasingly required for diagnosis, prognosis and treatment decision. Furthermore, molecular testing can provide genetic stratification for clinical trials and may guide clinical management.

In the present study, we validated a targeted-NGS method, which was adjusted in a diagnostic setting, and we applied it to analyze a cohort of 212 ADPKD patients. Our protocol is based on high-throughput simultaneous sequencing of *PKD1* and *PKD2* after LR-PCR of coding regions, followed by a masked reference genome alignment. Samples without *PKD1*/*PKD2* mutations were submitted to MLPA and, when negative, tested by targeted-NGS for mutations in newly described ADPKD and ADPLD genes, ARPKD and additional cystogenes.

Our protocol overcomes the technical issues of *PKD1* analysis, provides a reliable and less expensive molecular test for both genes, and resulted highly accurate. The use of previously validated primers for gene-specific enrichment, the introduction of an additional LR-PCR in order to minimize the risk of allele-drop out, and a specific alignment pipeline for avoiding miss-alignment with pseudogenes, make our genetic test specific and robust. Nevertheless, we cannot exclude that few variants were missed due to non-amplification of one allele.

Overall, we detected a pathogenic/likely pathogenic variant in 63.9% (122 among 191) of our index patients, 100 (82.0%) of which showed a genetic defect in *PKD1*, 17 (14%) in *PKD2* and 5 (4%) in other cystogenes. In addition, 26 probands (13.6%) presented variants in these genes classified as uncertain significance.

Several studies have demonstrated that patients with *PKD1* mutations predicted to truncate the protein have a more severe phenotype than patients with non-truncating mutations and patients with *PKD2* mutations. In addition, hypomorphic alleles are reported in patients with milder kidney disease and in normal individuals, but can cause a severe early onset disease if they are present in *trans* with a pathogenic variant. Hence, we also included gene and type of detected variant in the nosology of our patient cohort (Cornec-Le Gall et al., 2013; Heyer et al., 2016; Hwang et al., 2016). In our study, we detected a higher prevalence of ADPKD-*PKD1*^T^ cases in the confirmation cohort (89.3%) in comparison to that in the diagnostic cohort (76.4%), according to the different clinical phenotype of patients in the two groups. Moreover, our overall results confirm that patients with *PKD1* mutations predicted to truncate the protein experienced ESRD 9 years earlier than patients with *PKD1* non-truncating mutations and >13 years earlier than patients with *PKD2* mutations. In addition, our ADPKD-*PKD1*^T^ cases showed an onset of the disease significantly earlier than ADPKD-*PKD1*^NT^ and ADPK-*PKD2* (>10 and >19 years, respectively), as well as a significant earlier diagnosis (7 and 15 years, respectively). These data emphasize the need to combine clinical information with gene and allele data to achieve useful prognostic predictions. The incomplete penetrance of some PKD alleles and a dosage effect must be taken into account.

Among 158 distinct variants here detected, 80 (50.6%) were previously unreported, confirming the high allelic heterogeneity of these genes, which harbor many clinically significant private variants. Differentiating pathogenic from neutral changes remains a big challenge, since 36.7% of the variants here detected were non-truncating, 19% were classified as variants of uncertain significant and 5.1% were likely hypomorphic alleles. Our additional bioinformatics analysis using PyMOL helped us to better define the potential pathogenicity of some VUS and H variants. However, variant interpretation remains the major bottleneck in NGS analysis and data sharing is essential, in order to collect more families where segregation analysis can be performed. It is notable that, when strictly following ACMG guidelines, the only non-truncating *PKD1*/*PKD2* variants that reach the status of “likely pathogenic” are either described in previous studies or segregate in affected family members. We did our best to organize and perform segregation analysis in as many families as possible. Functional studies to evaluate *PKD1* and *PKD2* variants would be extremely useful but, unfortunately, are currently unavailable.

A further key point is the distinction between neutral and hypomorphic alleles and the degree of their penetrance. This is relevant in order to select potential kidney donors and to assess the risk of biallelic disease when a hypomorphic variant is present in *trans* with a fully inactivating *PKD1* allele. Indeed, some ADPKD and ARPKD patients can clinically overlap with early manifestation in ADPKD and late onset in ARPKD. Mutations in *PKD1* and *PKD2* can also be inherited in a recessive way with at least one *PKD1* or *PKD2* hypomorphic allele (Bergmann, 2017). In our study, eleven patients (9.4% of mutated) showed more than one variant. Segregation analysis indicated biallelic disease in five patients, digenic in one, *de-novo* variant with unknown phase in two, and segregation analysis was not possible in three patients. Seven patients with very early onset disease and 23 patients with early onset were present in our series, and 23.3% (7 out 30) of them exhibited two variants, typically compound heterozygosis of a pathogenic and a hypomorphic allele.

We found a further clinical phenocopy of ARPKD, due to homozygosity of the *PKD1* hypomorphic allele p.(Arg3277Cys), in a patient with negative family history (ID 17045). This variant is reported as an incompletely penetrant mutant allele that resulted in occasional cyst development in heterozygotes and more severe PKD in homozygosity (Rossetti et al., 2009). Our data confirmed that the dosage/threshold of functional *PKD1* protein might be critical for cyst initiation. Of note, the *in silico* analysis by PyMol indicated that this variant results in a bond loss in Polycystin-1.

A case of ADPKD_VEO_ caused by digenic disease with *PKD1* and *PKD2* genetic defects was also detected. Digenic disease involving *PKD1* and *PKD2* genes are rarely described, and it usually results in more severe clinical phenotype than in cases with mono-allelic variants, but not in VEO (Pei et al., 2001; Gainullin et al., 2015). The co-inheritance of a pathogenic allele in *PKD2* (from the affected mother) and a hypomorphic variant in *PKD1* from the unaffected father, likely caused a severe phenotype in this family. To our knowledge, this is the first report of bilineal inheritance causing prenatal onset of polycystic kidneys.

We found one patient with biallelic mutations in *PKHD1* among probands negative for *PKD1*/*PKD2* mutations, confirming the significant overlap between ARPKD and ADPKD. ARPKD is usually a severe early onset disease, but there is wide variability in severity even among patients carrying the same *PKHD1* mutations, emphasizing the importance of modifying genes and potential environmental factors (Gunay-Aygun et al., 2010). In a sporadic patient with adult-onset polycystic kidneys, the diagnosis of ARPKD should be considered. Such cases highlight the importance of the molecular diagnosis: knowing the genetic cause of the disease allows an adequate clinical management and a proper genetic counseling.

NGS has the advantage of deep sequencing and allows the detection of mosaicism, undetectable by Sanger sequencing. Somatic mutations acquired in the early stage of embryonic postzygotic development might cause mosaicism in the parent who later transmits the mutation to the offspring through germ cell. One case of mosaicism arising from this mechanism was detected in our study: the heterozygous *PKD1* mutation detected in the affected proband was inherited from the less severely affected mother, who showed only 6.8% of mutated reads in her peripheral blood. An additional case of somatic mosaicism has been detected, with 15.9% of mutated reads in *PKD1* gene. No offspring was available to test the heritability of this genetic defect. The incidence of mosaicism events in the human body is underestimated, but a mosaicism should be considered in patients with mild disease and healthy parents or with unclear pattern of transmission. In addition, apparently *de-novo* mutations might indeed be the consequence of somatic/germline mosaicism in an unaffected parent. In current diagnostic protocols, targeted deep sequencing of the mutation detected in the index case should be performed in the unaffected parents. Our NGS protocol represented a useful diagnostic tool for the detection of somatic mosaicism.

In conclusion, accurate and cost-effective diagnostic genetic tests are needed in order to drive clinical management of polycystic kidney disease. NGS technology represents a powerful approach for a better understanding of this disorder, overcoming at least part of the technical difficulties of *PKD2*/*PKD1* molecular analysis. However, interpretation of results remains challenging and requires interdisciplinary efforts as well as broad genetic and phenotypic data sharing.

## Data Availability Statement

The datasets generated for this study can be found in the https://databases.lovd.nl/shared/genes/PKD1; https://databases.lovd.nl/shared/genes/PKD2 (Fokkema et al., 2011).

## Ethics Statement

Ethical review and approval was not required for the study on human participants in accordance with the local legislation and institutional requirements. Written informed consent to participate in this study was provided by the participants' legal guardian/next of kin.

## Author Contributions

VM: study design, analysis and interpretation of experimental data, statistical analyses, manuscript preparation, and critical revision of the manuscript. SB: study design, technical analysis and interpretation of experimental data, and manuscript preparation. CG: patients' recruitment, clinical data collection and interpretation, genetic counseling, and manuscript preparation. IC: patients' recruitment and their clinical management, clinical data collection and interpretation, and manuscript preparation. RM, CC, MP, and SD: technical analysis and interpretation of experimental data. VA and RC: clinical data collection. EA: clinical data collection, genetic counseling. AM: technical analysis and interpretation of experimental data, and bioinformatics analyses. FF and EG: patients' recruitment, clinical data collection and interpretation, and manuscript preparation. EM, FM, and AP: patients' recruitment, clinical data collection and interpretation. NS: performed MRI of patients. AW and MS: patients' recruitment, clinical data collection and interpretation, and genetic counseling. GL: patients' recruitment and their clinical management, clinical data collection and interpretation, critical revision of the manuscript and corresponding author. All authors read and approved the manuscript for submission.

## Conflict of Interest

The authors declare that the research was conducted in the absence of any commercial or financial relationships that could be construed as a potential conflict of interest.

## Footnotes

^1^Integrative Genome Viewer (IGV). http://www.broadinstitute.org/igv

^2^Human Genome Variation Society. http://www.hgvs.org/mutnomen

^3^VarSomeClinical platform. https://varsome.com

^4^Mutation database PKDB. https://pkdb.mayo.edu

^5^Leiden Open Variations PKD database. https://databases.lovd.nl/shared/genes/PKD1; https://databases.lovd.nl/shared/genes/PKD2

^6^ClinVar. http://www.ncbi.nlm.nih.gov/clinvar

^7^PyMOL. www.pymol.org/2
