# Supplementary material for: Gene Panel Analysis in a Large Cohort of Patients With Autosomal Dominant Polycystic Kidney Disease Allows the Identification of 80 Potentially Causative Novel Variants and the Characterization of a Complex Genetic Architecture in a Subset of Families
Source: Front Genet. 2020 May 7;11:464. doi: 10.3389/fgene.2020.00464 (PMC7224062; doi:10.3389/fgene.2020.00464)
Supplement: Supplementary file 1 [file Data_Sheet_1.PDF]

Supplementary Material

1 Supplementary Tables

Supplementary Table 1. Oligonucleotide primers for eight and five long-range specific templates of *PKD1* (exons 1 to 46) and *PKD2* (exons 1-15), respectively. We combined previously described primers ([1]Tan et al., 2014; [2]Rossetti et al., 2012, [3]Kinoshita et al., 2016, [4]Audrézet et al., 2012) with primers designed by us[5], optimizing and validating the LR-PCR protocols. # New combination.
PCR amplification conditions are also described. For exon 15-21, 22-26 and 27-34 of *PKD1* a touch-down PCR protocol was performed. PCR enzyme and buffer used were GoTaq Long PCR Master Mix (Promega Corp., Madison, WI, USA), PrimeSTAR GXL DNA Polymerase (Takara Bio Inc., Shiga, Japan).

PKD1						
Exons covered	PCR primers (5'-3')	Target (kb)	Polymerase	Annealing temp. (°C)	Elongation (min)	Number of cycles
1	Fw CGCAGCCTTACCATCCACCT[1]<br>Rv TCATCGCCCCTTCCTAAGCA[1]	2,3	GoTaq	64	3	35
2-12	Fw CCAGCTCTCTGTCTACTCACCTCCGCATC[1]<br>Rv CCACGGTTACGTTGTAGTTCACGGTGACG[1]	8,7	PrimeSTAR	68	8.5	35
13-21	Fw TGGAGGGAGGGACGCCAATC[1]<br>Rv ACACAGGACAGAACGGCTGAGGCTA[1]	7,9	GoTaq	66	8.5	35
15-21	Fw AGCGCAACTACTTGGAGGCCC[2]<br>Rv GGAGCCCAGGCTGGAGGCTCA[2]	3,5	GoTaq	74 - 70 - 68	5 - 5 - 5	1+1+30
22-26	Fw ATGTGAAGAGGTGCCTTGTGTGGTCG[3]<br>Rv GCAATGAAGAGGAAAGCAGCACAGAG[3]	3,6	PrimeSTAR	74- 70 - 68	5 - 5 - 5	1+1+30
27-34	Fw GCCGGGACTGCCTGTGTGGCTCCTTGAGTGCGCAC[3]<br>Rv CTGGGGCCCTGGGGATCCCATGAGGCTCTTTCCA[3]	3,7	GoTaq	74 - 70 - 68	5 - 5 - 5	1+1+30
34-41#	Fw AGGAGGGGGCTCTGAAGCTCACCCTT[4]<br>Rv AGAGGGTGCGGGTCAGTAGG[4]	5,7	GoTaq	70	6	35

42-46	Fw CCAGGAGCCCACCCTCACTC[4]<br>Rv CCATTCTGCCTGGCCCTC[5]	2,1	GoTaq	62	3	35
PKD2						
Exons covered	PCR primers (5'-3')	Target (kb)	Polymerase	Annealing temp. (°C)	Elongation (min)	Number of cycles
1	Fw GTGGAGACAGAAGCCAACCAAAGAG[1]<br>Rv GGATGCGAGATGGAGCCCG[1]	1.4	GoTaq	64	3	35
2	Fw TTTCTTTCCATTTGCAATGTTTCATTC[1]<br>Rv GGAAGATAGTCAATAAACAAATGCCCAA[1]	2.5	PrimeSTAR	74- 70 - 68	5 - 5 - 5	1+1+30
3-6	Fw GAGAAGACCTTGTGTGAATTTGTCCA[1]<br>Rv TCATACTCAGCAAAGTTACTCATGCAAA[1]	10.9	PrimeSTAR	74- 70 - 68	5 - 5 - 5	1+1+30
7-10	Fw TCGGGTAAGTATAATGGTGAGCCCT[1]<br>Rv CATCAAGACTCCAAGATAGGGAACATTT[1]	10.3	PrimeSTAR	74- 70 - 68	5 - 5 - 5	1+1+30
11-15	Fw CACGTACTTGTTGAATGGCCAATGT[1]<br>Rv ATGAAACTCAGAAGCCCTTTGACAGTT[1]	10.8	PrimeSTAR	74- 70 - 68	5 - 5 - 5	1+1+30

Supplementary Table 2. Sensitivity, specificity and accuracy of NGS for PKD1 and PKD2 compared to Sanger sequencing. Data arise from validation cohort, where all mutations previously detected by Sanger sequencing were confirmed by NGS, as well as from the confirmation and diagnostic cohort, where all the possible pathogenic variants detected by NGS were submitted to Sanger sequencing. Sanger positive/negative: number of variants present/absent by Sanger sequencing. NGS positive/negative: variants present/absent by NGS method. Sensitivity was calculated as TP/TP+FN, specificity as TN/TN+FP and accuracy as TP+TN/TP+TN+FP+FN. Sanger method was considered as gold standard. T: true; P: positive; F: false; N: negative.

			Sensitivity %	Specificity %	Accuracy %
PKD1	Sanger positive	Sanger negative			
NGS positive	198 (TP)	21 (FP)			
NGS negative	0 (FN)	35 (TN)	100	63	92
PKD2	Sanger positive	Sanger negative			
NGS positive	23(TP)	1 (FP)			
NGS negative	0 (FN)	3 (TN)	100	75	96
PKD1/PKD2	Sanger positive	Sanger negative			
NGS positive	221 (TP)	22 (FP)			
NGS negative	0 (FN)	38 (TN)	100	63	92

Supplementary Table 3. Description of the 158 possible pathogenic variants detected in *PKD1* and *PKD2* genes. M: missense; F: frameshift; N: non-sense; IF= in-frame; S: splicing; LR: large rearrangement; T: truncating; NT: non truncating; H: hypomorphic; P: pathogenic; LP: likely pathogenc; VUS: variant of uncertain significant. ACMG: American College of Medical Genetics and Genomics standard. We performed ACMG classification by using the VarSomeClinical platform (https://varsome.com). We re-classified some variants after evaluation of databases (see Materials and methods) and thanks to segregation analysis in the families.

Gene	DNA change	Protein change	Exon	Number of families	Function	T/NT	ACMG classification/ Our re-classification	Described
PKD1	c.230A>G	p.Asn77Ser	2	1	M	NT	VUS/LP	Cornec-Le Gall et al., 2016
PKD1	c.301A>G	p.Asn101Asp	3	1	M	NT	VUS/LP	Leiden Open Variations PKD database
PKD1	c.368G>C	p.Ser123Thr	4	1	M	NT	LB/H	dbSNP database rs748717453
PKD1	c.484del	p.Ala162Leufs*128	4	1	F	T	P	This study
PKD1	c.803dup	p.Phe269Leufs*102	5	1	F	T	P	This study
PKD1	c.974A>G	p.Tyr325Cys	5	1	M	NT	LB/VUS	Rossetti et al., 2007
PKD1	c.1261C>T	p.Arg421Cys	6	1	M	NT	LB/VUS	Chang et al., 2013
PKD1	c.1295C>T	p.Ala432Val	6	2	M	NT	LP	Rossetti et al., 2007
PKD1	c.1376G>C	p.Arg459Pro	6	1	M	NT	LB/VUS	This study
PKD1	c.1477C>T	p.Gln493*	7	1	N	T	P	This study
PKD1	c.1569_1570del	p.Pro525Alafs*61	7	1	F	T	P	This study
PKD1	c.1682C>T	p.Ala561Val	8	1	M	NT	LB/H	This study
PKD1	c.1777G>A	p.Glu593Lys	9	1	M	NT	VUS	This study
PKD1	c.1832G>C	p.Arg611Pro	9	1	M	NT	VUS	This study
PKD1	c.2059_2064del	p.Leu687_Phe688del	10	1	IF	NT	VUS	This study
PKD1	c.2098-2_2109del	p.?	IVS10/ex11	1	F	T	LP	This study
PKD1	c.2180T>C	p.Leu727Pro	11	1	M	NT	LP	Rossetti et al., 2007
PKD1	c.2329C>T	p.Gln777*	11	1	N	T	P	Trujillano et al., 2014
PKD1	c.2438G>A	p.Cys813Tyr	11	1	M	NT	VUS	This study
PKD1	c.2494dupC	p.Arg832Profs*40	11	1	F	T	P	Rossetti et al., 2012
PKD1	c.2830C>T	p. Arg944Cys	11	1	M	NT	VUS	Kim et al., 2019
PKD1	c.2986-2_2987del	p.?	IVS12/ex13	1	F	T	LP	This study

PKD1	c.3161+1G>A	p.?	13	1	S	T	P	This study
PKD1	c.3210_3222del	p.Tyr1071Serfs*29	14	1	F	T	P	This study
PKD1	c.3236del	p.Asp1079Alafs*25	14	1	F	T	P	This study
PKD1	c.3250C>T	p.Gln1084*	14	1	N	T	P	PKDB mutation database
PKD1	c.3398_3399del	p.Val1133Glufs*2	15	1	F	T	P	This study
PKD1	c.3490G>A	p.Gly1164Arg	15	1	M	NT	VUS	Jin et al., 2016
PKD1	c.3554G>A	p.Gly1185Asp	15	1	M	NT	VUS/H	This study
PKD1	c.3706C>T	p.Gln1236*	15	1	N	T	P	Carrera et al., 2016
PKD1	c.3735_3741del	p.Phe1245Leufs*26	15	1	F	T	P	Leiden Open Variations PKD database
PKD1	c.4041_4042del	p.His1347Glnfs*83	15	1	N	T	P	Leiden Open Variations PKD database
PKD1	c.4168C>T	p.Gln1390*	15	1	N	T	P	This study
PKD1	c.4232_4239del	p.Arg1411Leufs*17	15	1	F	T	P	This study
PKD1	c.4429del	p.Leu1479Trpfs*55	15	1	F	T	P	This study
PKD1	c.4494C>G	p.Tyr1498*	15	1	N	T	P	This study
PKD1	c.4551C>A	p.Tyr1517*	15	1	N	T	P	Solazzo et al., 2018
PKD1	c.4573G>T	p.Val1525Phe	15	1	M	NT	VUS/LP	This study
PKD1	c.4631_4640del	p.Val1544Alafs*16	15	1	F	T	P	This study
PKD1	c.4797C>A	p.Tyr1599*	15	1	N	T	P	Xu et al., 2018
PKD1	c.4888C>T	p.Gln1630*	15	1	N	T	P	This study
PKD1	c.5014_5015del	Arg1672Glyfs*98	15	3	F	T	P	Watnick et al., 1999
PKD1	c.5102A>T	p.Asn1701Ile	15	1	M	NT	VUS/LP	This study
PKD1	c.5425del	p.Ala1809Profs*27	15	1	F	T	P	This study
PKD1	c.5482C>T	p.Gln1828*	15	2	N	T	P	Rossetti et al., 2002
PKD1	c.5511G>A	p.Trp1837*	15	1	N	T	P	Rossetti et al., 2002
PKD1	c.5536del	p.Ser1846Alafs*103	15	1	F	T	P	This study
PKD1	c.5622G>A	p.Trp1874*	15	1	N	T	P	Roelfsema et al., 1997
PKD1	c.5884C>T	Gln1962*	15	1	N	T	P	This study
PKD1	c.5989C>T	p.Gln1997*	15	1	N	T	P	This study
PKD1	c.5999C>G	p.Ser2000Cys	15	1	M	NT	LB/H	This study

PKD1	c.6016del	p.Trp2006Glyfs*110	15	2	F	T	P	This study
PKD1	c.6040C>T	p.Gln2014*	15	1	N	T	P	Inoue et al., 2002
PKD1	c.6184C>T	p.Gln2062*	15	1	N	T	P	This study
PKD1	c.6424C>T	p.Gln2142*	15	2	N	T	P	Yu et al., 2011
PKD1	c.6499A>G	p.Asn2167Asp	15	1	M	NT	VUS	This study
PKD1	c.6571C>T	p.Arg2191Cys	15	1	M	NT	VUS	dbSNP database rs375006983
PKD1	c.6730_6731del	Ser2244Hisfs*17	15	1	F	T	P	Yu et al., 2011
PKD1	c.6749C>T	p.Thr2250Met	15	1	M	NT	VUS/H	Perrichot et al., 2000
PKD1	c.6764G>C	p.Arg2255Pro	15	1	M	NT	VUS	This study
PKD1	c.7065+5G>T	p.?	IVS16	1	S	T	VUS	This study
PKD1	c.7126C>T	p.Gln2376*	17	1	N	T	P	Rossetti et al., 2002
PKD1	c.7137C>G	p.Tyr2379*	17	1	N	T	P	Audrézet et al., 2012
PKD1	c.7166T>C	p.Leu2389Ser	17	1	M	NT	VUS	This study
PKD1	c.7288C>T	p.Arg2430*	18	1	N	T	P	Phakdeekitcharoen et al., 2000
PKD1	c.7301_7312dup	Arg2434_Val2437dup	18	1	IF	NT	LP	This study
PKD1	c.7489+1G>A	p.?	IVS18	1	S	T	P	This study
PKD1	c.7535T>C	p.Leu2512Pro	19	1	M	NT	VUS	This study
PKD1	c.7546C>T	p.Arg2516Cys	19	1	M	NT	LP	Garcia-Gonzalez et al., 2007
PKD1	c.7619C>G	p.Pro2540Arg	19	1	M	NT	VUS	dbSNP database rs750752232
PKD1	c.7622C>T	p.Pro2541Leu	19	1	M	NT	VUS/LP	Cornec-Le Gall et al., 2016
PKD1	c.7663G>A	p.Val2555Met	19	2	M	NT	VUS/LP	This study
PKD1	c.7909C>T	p.Gln2637*	21	1	N	T	P	Irazabal et al., 2011
PKD1	c.7979A>G	p.Asp2660Gly	21	1	M	NT	VUS	This study
PKD1	c.7987C>T	p.Gln2663*	21	1	N	T	P	Audrézet et al., 2012
PKD1	c.8000C>A	p.Ala2667Glu	21	1	M	NT	VUS	This study
PKD1	c.8020C>T	p.Pro2674Ser	22	1	M	NT	LB/H	PKDB mutation database
PKD1	c.8179G>C	p.Ala2727Pro	23	1	M	NT	VUS	This study
PKD1	c.8270C>G	p.Ser2757Cys	23	1	M	NT	VUS	This study
PKD1	c.8311G>A	p.Glu2771Lys	23	4	M	NT	LP	Rossetti et al., 2001

PKD1	c.8428G>T	p.Glu2810*	23	1	N	T	P	Garcia-Gonzalez et al., 2007
PKD1	c.8560C>T	p.Gln2854*	23	2	N	T	P	Audrézet et al., 2012
PKD1	c.8824del	p.Leu2942Trpfs*52	24	1	F	T	P	This study
PKD1	c.8860G>T	p.Glu2954*	24	1	N	T	P	This study
PKD1	c.8935_8937del	p.Phe2979del	24	1	IF	NT	VUS/LP	Bouba et al., 2001
PKD1	c.8972dup	p.Tyr2991*	25	1	N	T	P	Audrézet et al., 2012
PKD1	c.9003G>A	p.Trp3001*	25	1	N	T	P	Leiden Open Variations PKD database
PKD1	c.9051C>A	p.Tyr3017*	25	1	N	T	P	This study
PKD1	c.9201+1G>A	p.?	IVS25	1	S	T	P	This study
PKD1	c.9240_9241del	Ala3082Cysfs*96	26	1	F	T	P	Thongnoppakhun et al., 2004
PKD1	c.9404C>T	p.Thr3135Met	27	1	M	NT	LP	Kurashige et al., 2015
PKD1	c.9499A>T	p.Ile3167Phe	27	1	M	NT	LB/H	Rossetti et al., 2002
PKD1	c.9547C>T	Arg3183*	27	1	N	T	P	Audrézet et al., 2012
PKD1	c.9553_9554del	p.Trp3185Alafs*32	27	1	F	T	P	This study
PKD1	c.9583T>G	p.Trp3195Gly	28	1	M	NT	VUS	This study
PKD1	c.9622del	p.Ala3208Hisfs*108	28	1	F	T	P	This study
PKD1	c.9713-1G>A	p.?	IVS28	1	S	T	P	This study
PKD1	c.9713-2A>G	p.?	IVS28	1	S	T	P	This study
PKD1	c. 9739C>T	p.Arg3247Cys	29	1	M	NT	LP	Kurashige et al., 2015
PKD1	c.9760C>T	p.Gln3254*	29	2	N	T	P	This study
PKD1	c.9829C>T	p.Arg3277Cys	29	1	M	NT	LB/H	Rossetti et al., 2009
PKD1	c.9852C>A	p.Cys3284*	29	1	N	T	P	This study
PKD1	c.9857T>C	p.Leu3286Pro	29	1	M	NT	VUS/LP	This study
PKD1	c.9965_9966del	Thr3322Serfs*67	30	1	F	T	P	This study
PKD1	c.9977G>A	p.Gly3326Asp	30	1	M	NT	VUS/LP	Neumann et al., 2013
PKD1	c.10219A>G	p.Ser3407Gly	32	1	M	NT	VUS	This study
PKD1	c.10700dup	p.Val3568Cysfs*59	36	1	F	T	P	This study
PKD1	c.10719_10720del	p.Gly3574Valfs*52	36	1	F	T	P	Audrézet et al., 2012
PKD1	c.10904del	p.Ala3635Valfs*2	37	1	F	T	P	This study

PKD1	c.11134del	p.Arg3712Glyfs*114	38	1	F	T	P	Turco et al., 1997
PKD1	c.11257_11269+3del	p.Arg3753fs	ex39/IVS39	1	F	T	P	This study
PKD1	c.11258G>A	p.Arg3753Gln	39	1	M	NT	LP	Rossetti et al., 2007
PKD1	c.11390A>G	p.Tyr3797Cys	40	1	M	NT	VUS	Carrera et al., 2016
PKD1	c.11417G>A	p.Trp3806*	41	1	N	T	P	Stekrova et al., 2009
PKD1	c.11424G>A	p.Trp3808*	41	1	N	T	P	This study
PKD1	c.11440T>C	p.Tyr3814His	41	1	M	NT	VUS	This study
PKD1	c.11450G>A	p.Gly3817Glu	41	1	M	NT	VUS	This study
PKD1	c.11512C>T	p.Gln3838*	41	1	N	T	P	Peral et al., 1996
PKD1	c.11614G>T	p.Glu3872*	42	1	N	T	P	Tan et al., 2009
PKD1	c.11635_11645del	p.Ala3879Profs*78	42	1	F	T	P	This study
PKD1	c.11819dup	p.Leu3941Alafs*20	43	1	F	T	P	This study
PKD1	c.11872G>C	p.Ala3958Pro	43	1	M	NT	LB/VUS	This study
PKD1	c.12010C>T	p.Gln4004*	44	1	N	T	P	Kim et al., 2019
PKD1	c.12011dup	p.Gln4005Alafs*152	44	2	F	T	P	Carrera et al., 2016
PKD1	c.12032A>T	p.Gln4011Leu	44	1	M	NT	VUS	Kurashige et al., 2015
PKD1	c.12168G>A	p.Trp4056*	45	1	N	T	P	This study
PKD1	c.12232_12241del	p.Glu4078Thrfs*117	45	1	F	T	P	This study
PKD1	c.12240G>A	p.Trp4080*	45	1	N	T	P	PKDB mutation database
PKD1	c.12308del	p.Ala4103Valfs*95	45	1	F	T	P	This study
PKD1	c.12389_12391del	p.Val4130del	45	1	IF	NT	VUS/LP	Garcia-Gonzalez et al., 2007
PKD1	c.12444G>C	p.Glu4148Asp	45	1	M	NT	VUS/LP	This study
PKD1	c.12460C>T	p.Arg4154Cys	46	1	M	NT	LB/VUS	Perrichot et al., 1999
PKD1	c.12682C>T	p.Arg4228*	46	1	N	T	P	Peral et al., 1996
PKD1	g.2088710-g.2135898	deletion 1-46	1 to 46	1	LR	T	P	Carrera et al., 2016
PKD1	g.2113161-g.2114925	deletion 11-12	11 to 12	1	LR	T	P	This study
PKD1	g.2100396-g.2104642	deletion 22-27	22 to 27	1	LR	T	P	This study
PKD1	g.2092954-g.2102256	deletion 26-38	26 to 38	1	LR	T	P	Rossetti et al., 2007
PKD1	g.2093544-g.2094013	deletion 36-37	36 to 37	1	LR	T	P	This study

PKD1	g.2092047-g.2092188	deletion 40	40	1	LR	T	P	Kinoshita et al., 2016
PKD2	c.540_555dup	Arg186Glyfs*32	1	1	F	T	P	This study
PKD2	c.916C>T	p.Arg306*	4	1	N	T	P	Veldhuisen et al., 1997
PKD2	c.974G>A	p.Arg325Gln	4	1	M	NT	LP	Rossetti et al., 2007
PKD2	c.992G>A	p.Cys331Tyr	4	1	M	NT	LP	This study
PKD2	c.1158T>A	p.Tyr386*	5	1	N	T	P	Aguiari et al., 1999
PKD2	c.1307_1311del	p.Phe436Cysfs*7	5	1	F	T	P	This study
PKD2	c.1319+1G>A	p.?	5	1	S	T	P	Torra et al., 2000
PKD2	c.1445T>G	p.Phe482Cys	6	2	M	NT	B/VUS	Dedoussis et al., 2008
PKD2	c.1449dup	p.Ile484Tyrfs*42	6	1	F	T	P	This study
PKD2	c.1609_1610del	p.Gln537Valfs*12	7	1	F	T	P	This study
PKD2	c.1837C>T	p.Gln613*	8	1	N	T	P	Robinson et al., 2012
PKD2	c.1960C>T	p.Arg654*	9	2	N	T	P	Irazabal et al., 2011
PKD2	c.2186T>A	p.Leu729Gln	11	1	M	NT	VUS	dbSNP database rs569788968
PKD2	c.2614C>T	p.Arg872*	14	1	N	T	P	Reynolds et al., 1999
PKD2	c.709+1G>A	p.?	IVS2	1	S	T	P	Veldhuisen et al., 1997
PKD2	c.1094+1delGTAA	p.?	IVS4	3	S	T	LP	This study
PKD2	c.1094+3_1094+6del	p.?	IVS4	1	S	T	LP	Magistroni et al., 2003
PKD2	g.88007668-g.88077777	deletion 1-15	1 to 15	2	LR	T	P	Hwang et al., 2016
PKD2	g.88056086-g88058103	deletion 8-9	8 to 9	2	LR	T	P	This study

Supplementary Table 4. Clinical characteristics, mutated genes and mutation types. SD: Standard Deviation; *p calculated by Chi-Square test; **p calculated by One-Way Analysis of Variance.

Clinical characteristics	N.	Yes (%)	Mutated gene vs clinical characteristics PKD1 vs PKD2 p*	Mutation type vs clinical characteristics $PKD1^T$ vs $PKD1^{NT}$ vs PKD2 p*
Positive family history	193	148 (76,7)	>0,05	>0,05
Presence of liver cysts	159	128 (80,5)	>0,05	>0,05
Vascular abnormalities	121	24 (19,8)	>0,05	>0,05
Urological events before 35yr	128	34 (26,6)	>0,05	0,02
Antihypertensive treatment before 35yr	133	54 (40,6)	>0,05	0,0008
ESRD	165	42 (25,5)	>0,05	>0,05
Transplantation	177	45 (25,4)	>0,05	0,0006

	N.	median	PKD1 mean ± SD	PKD2 mean ± SD	p**	$PKD1^T$ mean ± SD	$PKD1^{NT}$ mean ± SD	PKD2 mean ± SD	p**
Age at onset, yr	163	25	24,8 ± 15,4	39,7 ± 15,2	0,0004	20,1 ± 11,6	30,9 ± 17,4	39,7 ± 15,2	0,0000
Age at diagnosis, yr	156	41	39,7 ± 14,5	51,0 ± 14,7	0,0008	35,4 ± 13,1	42,5 ± 16,4	51,0 ± 14,7	0.0001
Age at ESRD, yr	42	47	48,6 ± 9,8	59,8 ± 8,6	0,04	46,0 ± 6,0	55,1 ± 14,3	59,8 ± 8,6	0,002
Serum creatinine at presentation, mg/dl	155	1,15	1,8 ± 1,7	1,6 ± 1,0	>0,05	1,7 ± 1,3	1,7 ± 2,1	1,6 ± 1,0	>0,05
GFR at presentation, ml/min	131	66	65,0 ± 31,6	57,3 ± 32,6	>0,05	65,2 ± 31,7	71,0 ± 32,2	57,3 ± 32,6	>0,05

Supplementary Figures

Supplementary Figure 1. Molecular visualization of uncertain and hypomorphic variants by using PyMOL (www.pymol.org/2/). A: bond loss for aminoacid p.(Ala3958Pro); B: bond loss for p.(Arg3277Cys) in Polycystin-1.

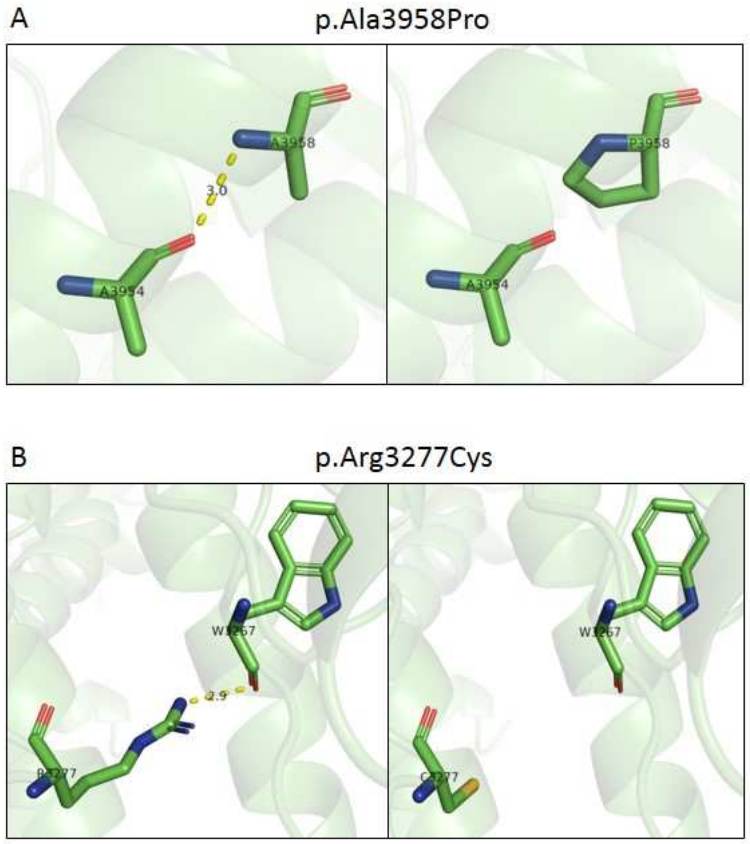

Supplementary References

Aguiari, G., Manzati, E., Penolazzi, L., Micheletti, F., Augello, G., Vitali, E. D. P., et al. (1999). Mutations in autosomal dominant polycystic kidney disease 2 gene: Reduced expression of PKD2 protein in lymphoblastoid cells. Am. J. Kidney Dis. 33, 880–885. doi:10.1016/S0272-6386(99)70420-8.

Bouba, I., Koptides, M., Mean, R., Costi, C. E., Demetriou, K., Georgiou, I., et al. (2001). Novel PKD1 deletions and missense variants in a cohort of Hellenic polycystic kidney disease families. Eur. J. Hum. Genet. 9, 677–684. doi:10.1038/sj.ejhg.5200696.

Chang, M. Y., Chen, H. M., Jenq, C. C., Lee, S. Y., Chen, Y. M., Tian, Y. C., et al. (2013). Novel PKD1 and PKD2 mutations in Taiwanese patients with autosomal dominant polycystic kidney disease. J. Hum. Genet. 58, 720–727. doi:10.1038/jhg.2013.91.

Dedoussis, G. V. Z., Luo, Y., Starremans, P., Rossetti, S., Ramos, A. J., Cantiello, H. F., et al. (2008). Co-inheritance of a PKD1 mutation and homozygous PKD2 variant: A potential modifier in autosomal dominant polycystic kidney disease. Eur. J. Clin. Invest. 38, 180–190. doi:10.1111/j.1365-2362.2007.01913.x.

Garcia-Gonzalez, M. A., Jones, J. G., Allen, S. K., Palatucci, C. M., Batish, S. D., Seltzer, W. K., et al. (2007). Evaluating the clinical utility of a molecular genetic test for polycystic kidney disease. Mol. Genet. Metab. 92, 160–167. doi:10.1016/j.ymgme.2007.05.004.

Inoue, S., Inoue, K., Utsunomiya, M., Nozaki, J. I., Yamada, Y., Iwasa, T., et al. (2002). Mutation analysis in PKD1 of Japanese autosomal dominant polycystic kidney disease patients. Hum. Mutat. 19, 622–628. doi:10.1002/humu.10080.

Jin, M., Xie, Y., Chen, Z., Liao, Y., Li, Z., Hu, P., et al. (2016). System analysis of gene mutations and clinical phenotype in Chinese patients with autosomal-dominant polycystic kidney disease. Sci. Rep. 6, 1–10. doi:10.1038/srep35945.

Kim, H., Park, H. C., Ryu, H., Kim, H., Lee, H. S., Heo, J., et al. (2019). Genetic Characteristics of Korean Patients with Autosomal Dominant Polycystic Kidney Disease by Targeted Exome Sequencing. Sci. Rep. 9, 1–12. doi:10.1038/s41598-019-52474-1.

Magistroni, R., He, N., Wang, K., Andrew, R., Johnson, A., Gabow, P., et al. (2003). Genotype-renal function correlation in type 2 autosomal dominant polycystic kidney disease. J. Am. Soc. Nephrol. 14, 1164–1174. doi:10.1097/01.ASN.0000061774.90975.25.

Peral B., San Millan J.L., Ong A.C.M., Gamble V., Ward C. J., Strong C., and H. P. C. (1996). Screening the 3' region of the polycystic kidney disease 1 (PKD1) gene reveals six novel mutations. Am J Hum Genet 58, 86–96. doi:10.1007/s004399900177.

Perrichot, R. A., Mercier, B., Simon, P. M., Whebe, B., Cledes, J., and Ferec, C. (1999). DGGE screening of PKD1 gene reveals novel

mutations in a large cohort of 146 unrelated patients. Hum. Genet. 105, 231–239. doi:10.1007/s004390051094.

Perrichot, R., Mercier, B., Quere, I., Carre, A., Simon, P., Whebe, B., et al. (2000). Novel mutations in the duplicated region of PKD1 gene. Eur. J. Hum. Genet. 8, 353–359. doi:10.1038/sj.ejhg.5200459.

Phakdeekitcharoen, B., Watnick, T. J., Ahn, C., Whang, D. Y., Burkhart, B., and Germino, G. G. (2000). Thirteen novel mutations of the replicated region of PKD1 in an Asian population. Kidney Int. 58, 1400–1412. doi:10.1046/j.1523-1755.2000.00302.x.

Reynolds, D. M., Hayashi, T., Cai, Y., Veldhuisen, B., Watnick, T. J., Lens, X. M., et al. (1999). Aberrant splicing in the PKD2 gene as a cause of polycystic kidney disease. J. Am. Soc. Nephrol. 10, 2342–2351.

Robinson, C., Hiemstra, T. F., Spencer, D., Waller, S., Daboo, L., Karet Frankl, F. E., et al. (2012). Clinical utility of PKD2 mutation testing in a polycystic kidney disease cohort attending a specialist nephrology out-patient clinic. BMC Nephrol. 13, 1–10. doi:10.1186/1471-2369-13-79.

Roelfsema, J. H., Spruit, L., Saris, J. J., Chang, P., Pirson, Y., Van Ommen, G. J. B., et al. (1997). Mutation detection in the repeated part of the PKD1 gene. Am. J. Hum. Genet. 61, 1044–1052. doi:10.1086/301600.

Rossetti, S., Strmecki, L., Gamble, V., Burton, S., Sneddon, V., Peral, B., et al. (2001). Mutation analysis of the entire PKD1 gene: Genetic and diagnostic. Am. J. Hum. Genet. 68, 46–63. doi:10.1086/316939.

Solazzo, A., Testa, F., Giovanella, S., Busutti, M., Furci, L., Carrera, P., et al. (2018). The prevalence of autosomal dominant polycystic kidney disease (ADPKD): A meta-analysis of European literature and prevalence evaluation in the Italian province of Modena suggest that ADPKD is a rare and underdiagnosed condition. PLoS One 13, 1–21. doi:10.1371/journal.pone.0190430.

Stekrova, J., Reiterova, J., Svobodova, S., Kebrdlova, V., Lnenicka, P., Merta, M., et al. (2009). New mutations in the PKD1 gene in Czech population with autosomal dominant polycystic kidney disease. BMC Med. Genet. 10. doi:10.1186/1471-2350-10-78.

Tan, Y. C., Blumenfeld, J. D., Anghel, R., Donahue, S., Belenkaya, R., Balina, M., et al. (2009). Novel method for genomic analysis of PKD1 and PKD2 mutations in autosomal dominant polycystic kidney disease. Hum. Mutat. 30, 264–273. doi:10.1002/humu.20842.

Thongnoppakhun, W., Limwongse, C., Vareesangthip, K., Sirinavin, C., Bunditworapoom, D., Rungroj, N., et al. (2004). Novel and de novo PKD1 mutations identified by multiple restriction fragment-single strand conformation polymorphism (MRF-SSCP). BMC Medical Genetics. 15, 1–15. doi:10.1186/1471-2350-5-2.

Torra, R., Badenas, C., Pérez-Oller, L., Luis, J., Millán, S., Nicolau, C., et al. (2000). Increased prevalence of polycystic kidney disease type 2 among elderly polycystic patients. Am. J. Kidney Dis. 36, 728–734. doi:10.1053/ajkd.2000.17619.

Turco, A. E., , Elena Bresin, S. R., Englisch, S., Pier Franco Pignatti, L. G., Maschio, G., Monica Bendetti, M. L. V., et al. (1997). Molecular Genetic Investigations in Autosomal Dominant Polycystic Kidney Disease. Contrib. Nephrol. 122, 53–57. doi:10.1016/B978-0-12-381462-3.00080-X.

Veldhuisen, B., Saris, J. J., De Haij, S., Hayashi, T., Reynolds, D. M., Mochizuki, T., et al. (1997). A spectrum of mutations in the second gene for autosomal dominant polycystic kidney disease (PKD2). Am. J. Hum. Genet. 61, 547–555. doi:10.1086/515497.

Watnick, T., Phakdeekitcharoen, B., Johnson, A., Gandolph, M., Wang, M., Briefel, G., et al. (1999). Mutation detection of PKD1 identifies a novel mutation common to three families with aneurysms and/or very-early-onset disease. Am. J. Hum. Genet. 65, 1561–1571. doi:10.1086/302657.

Xu, D., Ma, Y., Gu, X., Bian, R., Lu, Y., Xing, X., et al. (2018). Novel mutations in the PKD1 and PKD2 genes of Chinese patients with autosomal dominant polycystic kidney disease. Kidney Blood Press. Res. 43, 297–309. doi:10.1159/000487899.

Yu, C., Yang, Y., Zou, L., Hu, Z., Li, J., Liu, Y., et al. (2011). Identification of novel mutations in Chinese Hans with autosomal dominant polycystic kidney disease. BMC Med. Genet. 12. doi:10.1186/1471-2350-12-164.

Websites URLs

dbSNP database. https://www.ncbi.nlm.nih.gov/projects/SNP

Leiden Open Variations PKD database. https://databses.lovd.nl/shared/genes/PKD1

PKDB mutation database. https://pkdb.mayo.edu
